# Glycosylation and functionality of recombinant β-glucocerebrosidase from various production systems

**DOI:** 10.1042/BSR20130081

**Published:** 2013-09-25

**Authors:** Yoram Tekoah, Salit Tzaban, Tali Kizhner, Mariana Hainrichson, Anna Gantman, Myriam Golembo, David Aviezer, Yoseph Shaaltiel

**Affiliations:** *Protalix Biotherapeutics, 2 Snunit Street, P. O. Box 455, Carmiel, 2161401, Israel; †Faculty of Life Sciences, Bar Ilan University, Ramat Gan, 5290002, Israel

**Keywords:** ERT, Gaucher disease, glycosylation, lysosomal storage disease, macrophage, β-glucocerebrosidase, 2AB, 2-aminobenzamide, 4MU-G, 4-methylumbelliferyl β-D-glucopyranoside, DAPI, 4′,6-diamidino-2-phenylindole, CHO, Chinese-hamster ovary, ERT, enzyme replacement therapy, GD, Gaucher disease, GU, glucose unit, HSD, honestly significant difference, ICR, imprinting control region, MALDI–TOF-MS, matrix-assisted laser desorption ionization–time-of-flight-MS, MBL, mannose-binding lectin, MR, mannose receptor, NP, normal phase, PNGase A, peptide N-glycosidase A, PNGase F, peptide N-glycosidase F, PNP-G, *p*-nitrophenyl β-D-glucopyranoside, SA, sialic acid

## Abstract

The glycosylation of recombinant β-glucocerebrosidase, and in particular the exposure of mannose residues, has been shown to be a key factor in the success of ERT (enzyme replacement therapy) for the treatment of GD (Gaucher disease). Macrophages, the target cells in GD, internalize β-glucocerebrosidase through MRs (mannose receptors). Three enzymes are commercially available for the treatment of GD by ERT. Taliglucerase alfa, imiglucerase and velaglucerase alfa are each produced in different cell systems and undergo various post-translational or post-production glycosylation modifications to expose their mannose residues. This is the first study in which the glycosylation profiles of the three enzymes are compared, using the same methodology and the effect on functionality and cellular uptake is evaluated. While the major differences in glycosylation profiles reside in the variation of terminal residues and mannose chain length, the enzymatic activity and stability are not affected by these differences. Furthermore, the cellular uptake and in-cell stability in rat and human macrophages are similar. Finally, *in vivo* studies to evaluate the uptake into target organs also show similar results for all three enzymes. These results indicate that the variations of glycosylation between the three regulatory-approved β-glucocerebrosidase enzymes have no effect on their function or distribution.

## INTRODUCTION

GD (Gaucher disease) is a rare genetic lysosomal storage disorder caused by the functional deficiency of β-glucocerebrosidase that results in multiple organ malfunction [1]. β-glucocerebrosidase catalyses the hydrolysis of glucocerebroside into ceramide and glucose. In GD, the enzyme malfunction results in accumulation of excessive glucocerebroside in lysosomal compartments of tissue macrophages (Gaucher cells) [2]. A number of attempts to treat GD with intravenous ERT (enzyme replacement therapy) were made in the 1970s. The results were disappointing [3] with limited therapeutic value, because exogenously administered, unmodified human β-glucocerebrosidase, derived from placenta, did not effectively enter the target cells in the body, due to the lack of exposed mannose residues. Sequential removal of terminal sialic acid, galactose and *N*-acetyl-glucosamine sugars helped in targeting the MR (mannose receptor)-mediated endocytotic system of macrophages [4].

In order to produce β-glucocerebrosidase with exposed mannose residues, three alternative production methods have been developed and are used today for ERT of GD. Imiglucerase (Genzyme Corporation, a Sanofi company) is a β-glucocerebrosidase produced in a mammalian CHO (Chinese-hamster ovary) system, and modified post-production with exoglycosidase enzymes to expose mannose residues [5–7]. A gene-activated human β-glucocerebrosidase, velaglucerase alfa (Shire Pharmaceuticals Inc.) is produced in a human fibroblast carcinoma cell line and uses kifunensine, a mannosidase I inhibitor, in the medium during production to obtain the desired glycosylation profile. The inhibition of the natural maturation of the glycans generates, predominantly, high-mannose-type oligosaccharides [8]. Taliglucerase alfa (Protalix Biotherapeutics) is produced using a novel ProCellEx® plant cell-based protein expression system. This platform utilizes genetically modified carrot cells expressing the enzyme with the appropriate terminal mannose residues [9]. Terminal paucimannosidic type *N*-glycans are achieved by targeting to the plant storage vacuoles, where the terminal residues are removed [10].

The natural human β-glucocerebrosidase is composed of 497 amino acids and has five potential glycosylation sites, of which the first four are usually occupied [11] and all three commercial enzyme sequences incorporate all these glycosylation sites. Taliglucerase alfa differs from imiglucerase and velaglucerase alfa as it has two additional residues at the N terminus (EF) and seven additional residues at the C terminus (DLLVDTM) [9]. Limited reports have been published comparing the various preparations of recombinant β-glucocerebrosidase. Shaaltiel et al. [9] compared taliglucerase alfa and imiglucerase, and showed that the enzymes display highly homologous, three-dimensional structures, similar levels of biological activity and increased uptake of taliglucerase alfa into macrophages. Brumshtein et al. [8] compared imiglucerase and velaglucerase alfa, and concluded that both have similar three-dimensional structures and comparable kinetic parameters, and a two-fold greater rate of internalization into a human macrophage cell line (U937) for velaglucerase alfa. A study by Berger et al. [12] suggests lower uptake of taliglucerase alfa into blood monocytes from healthy donors and GD patients when compared with imiglucerase and velaglucerase alfa. In addition, Van Patten et al. [13] explored the correlation between the mannose chain length and the affinity to MRs. The authors concluded that MR binding, macrophage uptake and *in vivo* targeting were not affected by the type of glycoforms present, except for increased binding to the circulating MBL (mannose-binding lectin) by high-mannose glycoform. A comparison of the three production systems can also be found in Table 1.

**Table 1:** Summary of main glycan structures in three β-glucocerebrosidase enzymes CHO, Chinese-hamster ovary; Man_x_, amount of mannose sugars on glycans; ND, not detected.

Property	Taliglucerase alfa	Imiglucerase	Velaglucerase alfa
Engineered cells	Carrot cells	CHO cells	human fibrosarcoma cells
Amino acid sequence	R 495H EF at N-terminal; DLLVDTM at C-terminal	R495H	Natural human sequence
Method used to produce required glycans	Targeting to vacuole to naturally produce pauci-mannose structures	Exoglycosidase digestion (post-production) to expose mannose sugars	Addition of kifunensine to inhibit glycosylation process and produce high mannose structures
Exposed mannose	~100%*	40–60%	~100%†
Mannose chain length	Man_3_	Man_3_	Man_5_–Man_9_
Additional sugars	Xylose and/or core (α1-3) fucose	GlcNAc/Gal/SA and core (α1-6) fucose	ND

*All are paucimannose structures.

^†^All are high mannose structures.

The currently available data are controversial regarding the relative functionality of the enzymes. This is the first study to compare side-by-side, the effect of different post-translational modifications of *N*-glycosylation on *in vitro* and *in vivo* performance of the enzymes.

## MATERIALS AND METHODS

### Materials

Drug product of β-glucocerebrosidase from three manufacturers was used for all studies: taliglucerase alfa (Elelyso) was produced by Protalix Biotherapeutics, imiglucerase (Cerezyme, Genzyme Corporation) and velaglucerase alfa (VPRIV, Shire Pharmaceuticals). All drug products were prepared according to manufacturers’ instructions and recommendations. Reagents for SDS/PAGE were from Bio-Rad. Enzymes for glycan analysis were purchased from Prozyme. Water used throughout was either reverse-osmosis purified or HPLC grade (J.T. Baker). Human plasma was obtained from Bioreclamation LLC. Cell lines were purchased from the A.T.C.C. All other reagents and enzymes were purchased from Sigma-Aldrich, unless otherwise indicated.

### Glycosylation analysis of recombinant β-glucocerebrosidase enzymes

Enzymes (~200 μg) were separated on SDS/PAGE following reduction and alkylation. *N*-linked glycans were released from gel slices by incubation with Trypsin (Roche Diagnostics GmbH), followed by PNGase A (peptide N-glycosidase A) or by incubation with PNGase F (peptide N-glycosidase F) [14]. The glycans were fluorescently labelled with 2AB (2-aminobenzamide) and run on NP (normal phase) HPLC (Waters). Simultaneous exoglycosidase sequencing of the released glycan pool was performed as described previously [9,15]. The retention times of the individual glycans were compared with those of a standard partial hydrolysate of dextran, giving a ladder of GUs (glucose units). The following exoglycosidases were used: *Xanthomonas* β-1,2-xylosidase (Calbiochem), *Arthrobacter ureafaciens* sialidase, recombinant *Streptococcus pneumoniae* sialidase, bovine testes β-galactosidase, bovine kidney α-fucosidase, Jack bean β-hexosaminidase and Jack bean α-mannosidase.

Underivatized glycans were further purified using graphitized carbon tips (Hypercarb™, HyperSep™ tips, Thermo Fisher Scientific) according to the procedure recommended by the supplier. After the peptides were absorbed to the tips, the glycan-containing solution was dried in a centrifuge evaporator and re-suspended in 1–5 μl water. Samples (1 μl) were mixed with a similar volume of saturated solution (about 20 mg/ml) of 2,5-dihydroxybenzoic acid (Bruker-Daltonics) in acetonitrile/water, (1:1 v/v). The mixture (1 μl) was spotted on the MALDI (matrix-assisted laser desorption-ionization) target and allowed to dry at room temperature. When needed, samples were diluted with water before spotting. MALDI–TOF (time-of-flight)-MS was performed on a Bruker-Daltonics Reflex IV mass spectrometer at the Ilse Katz Institute for Nanoscale Science and Technology at Ben-Gurion University, Beer-Sheva, Israel. A nitrogen laser of 337 nm was used. Spectra were run in a positive mode in a linear flight path with an extraction voltage of 20 kV. At least 400 random shots across the sample were taken. The instrument was calibrated with standard I (Bruker-Daltonics) containing compounds with molecular masses ranging from 3 to17 kDa. X-Mass software was used for the analysis. Monoisotopic masses of the [M+Na]^+^ ions were within 0.1 mass units of the calculated values.

### MALDI–TOF-MS of intact protein and glycan site occupancy

Reconstituted protein samples (1 μl) were mixed with 9 μl of a saturated matrix sinapinic acid in a solution using 0.1% trifluoroacetic acid (J.T. Baker) and acetonitrile (J.T. Baker) as solvents, at a ratio of 2:1 (v/v). The mixture (1 μl) was spotted on the MALDI target and allowed to dry at room temperature. MALDI–TOF-MS analysis was performed on a Bruker-Daltonics Reflex IV mass spectrometer at the Ilse Katz Institute for Nanoscale Science and Technology at Ben-Gurion University, Beer-Sheva, Israel. A nitrogen laser of 337 nm was used. Spectra were run in a positive mode in a linear flight path with an extraction voltage of 20 kV. At least 300 random shots across the sample were taken. The instrument was calibrated with standard protein mix II (Bruker-Daltonics) containing compounds with molecular masses ranging from 20 to 70 kDa. FlexAnalysis software was used for the analysis. The average mass from the main singly charged monomer peak was taken for further calculation of the glycan site occupancy. Additional measurements for taliglucerase alfa were conducted on a Perseptive Biosystems Voyager STR DE-MALDI–TOF-MS at SGS M-Scan Ltd. using a similar method as above with minor modifications. In short, 1 μl protein samples were mixed with 1 μl matrix solution and allowed to air dry. Measurement was performed in linear mode. The instrument was calibrated with a mixture of myoglobin/BSA as a standard (1:20–1:100 respective protein ratios).

Glycan site occupancy was calculated by comparing the measured molecular mass of the protein to the theoretical mass, without the glycans. The difference in mass was divided by the weighted average of the glycans present on each specific enzyme, to give the average site occupancy.

### Enzymatic activity with synthetic substrates

Kinetic parameters for taliglucerase alfa, imiglucerase and velaglucerase alfa were measured by endpoint kinetics based on the hydrolysis of either PNP-G (*p*-nitrophenyl β-D-glucopyranoside) or 4MU-G (4-methylumbelliferyl β-D-glucopyranoside). In both cases, the reaction was initiated by the addition of 10 μl of diluted enzyme to an activity buffer as detailed below (final concentration of 0.1 μg/ml) and the concentration of either phenolate or 4MU products was obtained quantitatively based on an appropriate calibration curve. The parameters *K*_M_ and *V*_max_ were calculated by non-linear fit using GraFit software (Erithacus Software). Both reactions were incubated for 45 min at 37°C. PNP-G concentrations were 0.5–20 mM using an activity buffer (20 mM citrate, 30 mM sodium phosphate, 0.125% taurocholic acid, 1.5% Triton X-100 pH 5.5). After incubation, the reaction was quenched with 20 μl of 5 N NaOH and the absorbance of the phenolate product was read at a wavelength of 405 nm (TECAN Infinite M200). The concentrations for 4MU-G were 0.1–10 mM using an appropriate activity buffer (50 mM citrate, 176 mM potassium phosphate, 10 mM taurocholic acid, 0.01% Tween 20, pH 5.5). After incubation, 10 μl aliquots from the reaction mixture were added to 90 μl of stop solution (1M NaOH, 1M glycine, pH 10) and the fluorescence was measured at excitation/emission of 370/440 nm (TECAN Infinite M200).

### Enzymatic activity with the natural substrate

Endpoint kinetics of hydrolysis of the native substrate glucosylceramide (Matreya LLC) was performed. Seventy-six μl of the 10 mg/ml substrate stock solution in cloroform:methanol (2:1) were dried under vacuum using a CentriVap concentrator (Labconco) and re-dissolved in 4MU-G activity buffer (see above) to prepare a stock solution of 1000 μM. This stock solution was further diluted in the activity buffer to give concentrations ranging from 40 to 750 μM in a total volume of 50 μl. The reaction was initiated by the addition of 2 μl of diluted enzyme (115 ng/ml) to each preparation. The reaction was quenched after incubation (30 min, 37°C) by boiling (3 min, 100°C). The samples were cooled to room temperature and centrifuged for 5 min at 2000 rev./min. The resulting reaction mixtures were analysed for their glucose content using Glucose Assay Kit (Biovision), according to the manufacturer's instructions with minor modifications. In brief, glucose assay buffer (23.8 μl), enzyme mixture (1 μl) and fluorescent probe (0.2 μl) were mixed 1:1 with the tested solution and incubated for 1 h at 37°C. The fluorescence was read (Ex/Em=535/587 nm) using a multimode microplate reader (TECAN Infinite M200).

### Enzyme stability within biological matrices

The three tested enzymes were incubated at 37°C at a concentration of 2 μg/ml for either 20 min in human plasma (pH 7.4) or 4 days in a citrate-phosphate buffer (pH 4.6). Samples were evaluated for enzymatic activity at the various time points using the fluorimetric method with the synthetic substrate 4MU-G (see above) [16]. Results are expressed as a percentage of residual activity from the initial time point.

### Differentiation of human U937 macrophage cells

Human monocyte cell line U937 was optimally differentiated into macrophages by the addition of 75 ng/ml PMA [8] to the monocyte culture for 3 days (in 75 cm^2^ flasks). Macrophages were enriched by adhesion to the culture plate and identified by morphology and receptor staining.

### Flow cytometry of U937 cells before and after differentiation

Cells (0.5–1×10^6^) were blocked with 0.5% BSA in PBS for 10 min and stained with 1 μg/ml APC (allophycocyanin)-conjugated anti-CD11c (clone: 3.9; BioLegend). The cells were fixed with 0.5% formaldehyde and analysed in BD FACSCalibur™ flow cytometer (Becton Dickinson Biosciences) using a 655 nm filter.

### Uptake studies into macrophages

U937 cells, after differentiation (~1.6*10^6^ cells), were incubated with 60 μg/ml of the enzymes in F12K medium for 10 min at 5% CO_2_, 37°C incubator. The cells were then washed twice with ice cold PBS enriched with Mannan (1 mg/ml). This step is used to inhibit the uptake of the enzyme molecules through the MR [17]. To further ensure release of any proteins bound non-specifically to the membrane half of the cells were washed with ice cold glycine buffer: (0.8% NaCl, 0.038% KCl, 0.01% MgCl2, 0.01% CaCl2, 0.7% glycine [pH 3]) [18] followed by two additional cold PBS washes. The other half of the wells were used as controls for measuring total enzymatic activity (bound and internalized enzyme). The cells were lysed by adding β-glucocerebrosidase activity buffer (60 mM phosphate-citrate buffer; 0.15% Triton X-100; 0.125% sodium taurocholate pH 5.5), followed by pipetting and one freeze/thaw cycle.

The obtained lysates were subjected to determination of enzymatic activity using the colorimetric method detailed above. The total enzymatic activity was normalized to total soluble proteins as measured by Bradford assay. The effective uptake was calculated as the percentage of activity within the cells out of the total enzymatic activity of the controls.

### Immunofluorescence staining of MR in rat alveolar macrophages

Rat alveolar macrophages (line NR8383) were fixed with 4% PFA (paraformaldehyde) for 20 min. The cells were then permeabilized with 0.01% Triton-X-100 for 5 min at room temperature, and blocked with 5% BSA for 1 h. The MR was stained with polyclonal anti-MR polyclonal antibody (Abcam) at 100 μg/ml and Alexa Fluor 594 conjugated F(ab')_2_ fragment of goat anti-rabbit IgG (Life Technologies). Nuclei were stained using DAPI (4′,6-diamidino-2-phenylindole). The slides were analysed using a LSM 510 Meta laser scanning confocal system with a ×63/1.4 DIC oil objective using the 510 LSM software imaging system equipped with AxioCam digital microscope camera (Zeiss).

### In-cell stability of glucocerebrosidase in NR8383 cells

In order to evaluate the intracellular stability (half-life) of the enzymes after uptake into NR8383 rat alveolar macrophages, cells were incubated in 96-well, u-shaped plates (Nunc) in complete growth medium (F12K, Biological Industries). The enzymes (60 μg/ml) were added to the cells and incubated for 120 min at 5% CO_2_, 37°C to allow full protein uptake. The cells were washed extensively with PBS enriched with Mannan (1 mg/ml), and fresh complete growth medium (F12K) was added, for further incubation (up to 56 h). At each determined time point (3, 6, 16, 24, 48, 56 h), the cells were washed as described for U937, and lysed with ice-cold activity buffer. β-glucocerebrosidase activity was measured using the colorimetric method with synthetic substrate PNP-G. Total soluble proteins were measured by Bradford assay.

### Uptake into organs

#### Animals

Male ICR (imprinting control region; CD-1®) mice 6–8 weeks old (Harlan Laboratories) were handled according to the US NIH (National Institutes of Health) and the AAALAC (Association for Assessment and Accreditation of Laboratory Animal Care) guidelines. Animals were housed in polysulfone cages in a controlled environment with constant temperature (20–24°C) and a 12 h light/dark cycle, having *ad libitum* access to a commercial rodent diet (Teklad Certified Global 18% Protein Diet cat #: 106S8216) and drinking water.

#### Single infusion of three recombinant β-glucocerebrosidases

Taliglucerase alfa, imiglucerase and velaglucerase alfa were administered to the mice by single bolus injections, into one of the tail veins (intravenous), either at 5 mg/kg or 1.8 mg/kg. Groups of six rodents per enzyme were killed at 20, 40 or 60 min, post injection. Spleen, liver, lung and kidney tissues were collected and frozen at −70°C, until use. Organs were lysed with extraction buffer (20 mM phosphate buffer pH 7.2, 20 mM EDTA, 20 mM L-ascorbic acid, 1% Triton X-100 at a ratio of tissue:buffer, 1:5) in a TissuLyser (Qiagen), and assessed for enzymatic activity using a synthetic substrate 4MU-G as mentioned above.

#### Data analysis

Differences between measurements were assessed using one-way ANOVA Tukey–Kramer HSD (honestly significant difference) analysis (JMP 10.0.0, 2012 SAS institute Inc.), with *P* values <0.05 considered significant.

## RESULTS

### Glycosylation of β-glucocerebrosidase enzymes

**Figure 1:**
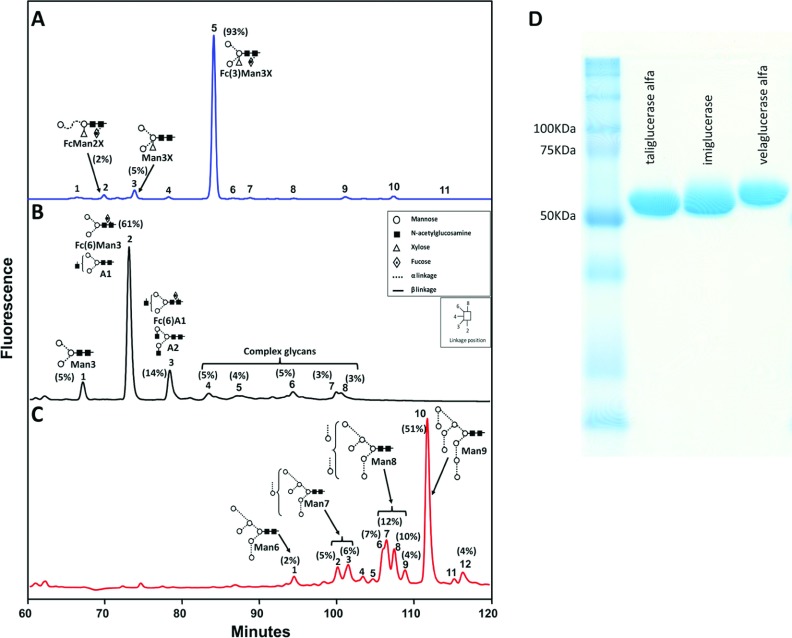
Comparison of the glycosylation profile of velaglucerase alfa, taliglucerase alfa and imiglucerase Representative NP-HPLC separation of glycans released by endoglycosidase digestion followed by fluorescent labelling, using 2AB. The relative area of the various glycans (above 2%) is indicated in parentheses, adjacent to the peak numbers. Main peaks in profiles are annotated with glycan structures and acronyms: Ax=(GlcNAc_x_)Man_3_-GlcNAc_2_; Fc=core fucose; Fc(3)=α1-3 linked fucose; Fc(6)=α1-6 linked Fucose; Man=mannose; Manx=Man_x_-GlcNAc_2_; X=xylose. (**A**) taliglucerase alfa shows a main peak of a paucimannose glycan Fc(3)Man_3_X; (**B**) imiglucerase shows a main peak of a mixture of Fc(6)Man_3_ and a mono-antennary (A1) structure, with further various complex glycans; and (**C**) velaglucerase alfa shows mainly variations of high mannose structure glycans (Man_3_-Man_9_); (**D**) SDS/PAGE separation of the various enzymes can be found to the left of the HPLC runs. Differences in retention time of bands on the gel correlate to the difference in molecular mass and glycosylation of the various enzymes. Further information can be found in Supplementary Table S1.

The different production systems of taliglucerase alfa, imiglucerase and velaglucerase alfa are expected to generate diverse glycosylation patterns. In order to analyse the glycan composition, enzymes were run on SDS/PAGE and the bands were extracted for further glycan analysis. The protein bands of each enzyme were comparable, at about 60 kDa, with the taliglucerase alfa sample showing a slightly lighter protein band, as expected, due to smaller plant glycosylation (Figure 1).

PNGase A that releases all glycans, including those containing plant derived, (α1-3)-linked fucose, was used to release glycans from taliglucerase alfa gel bands. The NP-HPLC profiles of the fluorescently labelled *N*-glycan pool from a representative taliglucerase alfa batch, released by PNGase A are shown in Figure 1(A). An additional digestion using PNGase F that releases only glycans that do not contain (α1-3)-linked fucose was performed in order to enable proper peak assignment. Final assignment of peaks was achieved by additional digestions using specific exoglycosidases (results not shown), by confirmation using MALDI-MS (Supplementary Table S1 available at http://www.bioscirep.org/bsr/033/bsr033e071add.htm) and by comparing GU values to known databases (http://glycobase.nibrt.ie/glycobase.html). The relative amounts of the main annotated glycans are listed in Figure 1.

Taliglucerase alfa shows a main peak containing a paucimannose structure (Man_3_GlcNAc_2_) with a (β1-2) xylose attached to the bisecting mannose and an (α1-3)-fucose attached to the reducing GlcNAc (*N*-acetylglucosamine), or in short, Fc(3)Man_3_X which comprises about 90% of the total glycan structures in this enzyme (Figure 1A). Digestion of taliglucerase alfa using PNGase F only released negligible amounts of glycans, demonstrating that the majority of the structures included glycans containing (α1-3)-linked fucose (results not shown).

The glycans from imiglucerase and velaglucerase alfa were released from gel bands by using only PNGase F, because they were not expected to contain (α1-3)-linked fucose. The NP-HPLC profiles of the fluorescently labelled *N*-glycan pool from representative batches of imiglucerase and velaglucerase alfa can be seen in Figures 1(B) and 1(C), respectively. As shown above, the final peak assignment was achieved by additional digestions using specific exoglycosidases (results not shown) and by confirmation using MALDI–TOF-MS (Supplementary Table S1).

The NP-HPLC profile of the glycans from imiglucerase shows a predominant peak of about 61%, which has been identified as Man_3_GlcNAc_2_ with an addition of either a core (α1-6)-linked fucose attached to the reducing GlcNAc, or an additional terminal GlcNAc. The remaining additional glycans (about 40%) are of various complexities, containing additional GlcNAc, Gal (galactose) and SA (sialic acid) residues at the non-reducing end of the glycans (Figure 1B).

Velaglucerase alfa shows a variety of high-mannose structures (Man_6–9_GlcNAc_2_) with the Man_9_GlcNAc_2_ being the predominant glycan (about 50%). The smaller high-mannose structures (Man_6–8_GlcNAc_2_) account for over 40% of the glycans and the remaining, about 10%, are of the complex-type structures (unassigned peaks in Figure 1C).

The relative percentage of the glycans released from all three β-glucocerebrosidase enzymes are indicated for all peaks, 2% or above, of the total released glycans.

### Molecular mass and glycan occupancy

Numerous measurements of intact molecular mass (total of 85) using MALDI–TOF-MS showed that taliglucerase alfa was found to have an average molecular mass of 60751 kDa, with a range of ±352 Da (×3 S.D.). Current measurement of imiglucerase gave a measurement of 60349 kDa, while measurement of velaglucerase alfa showed a higher molecular mass of 62519 kDa. In addition, based on the results of the glycan analysis, the calculated weighted average glycan mass was found to be 1160 Da and 1161 Da for taliglucerase alfa and imiglucerase, respectively, while the calculated weighted average glycan mass for velaglucerase alfa was 1790 Da. These data give an average glycan site occupancy of four glycosylation sites for all enzymes.

### *In vitro* enzymatic activity and stability

**Figure 2:**
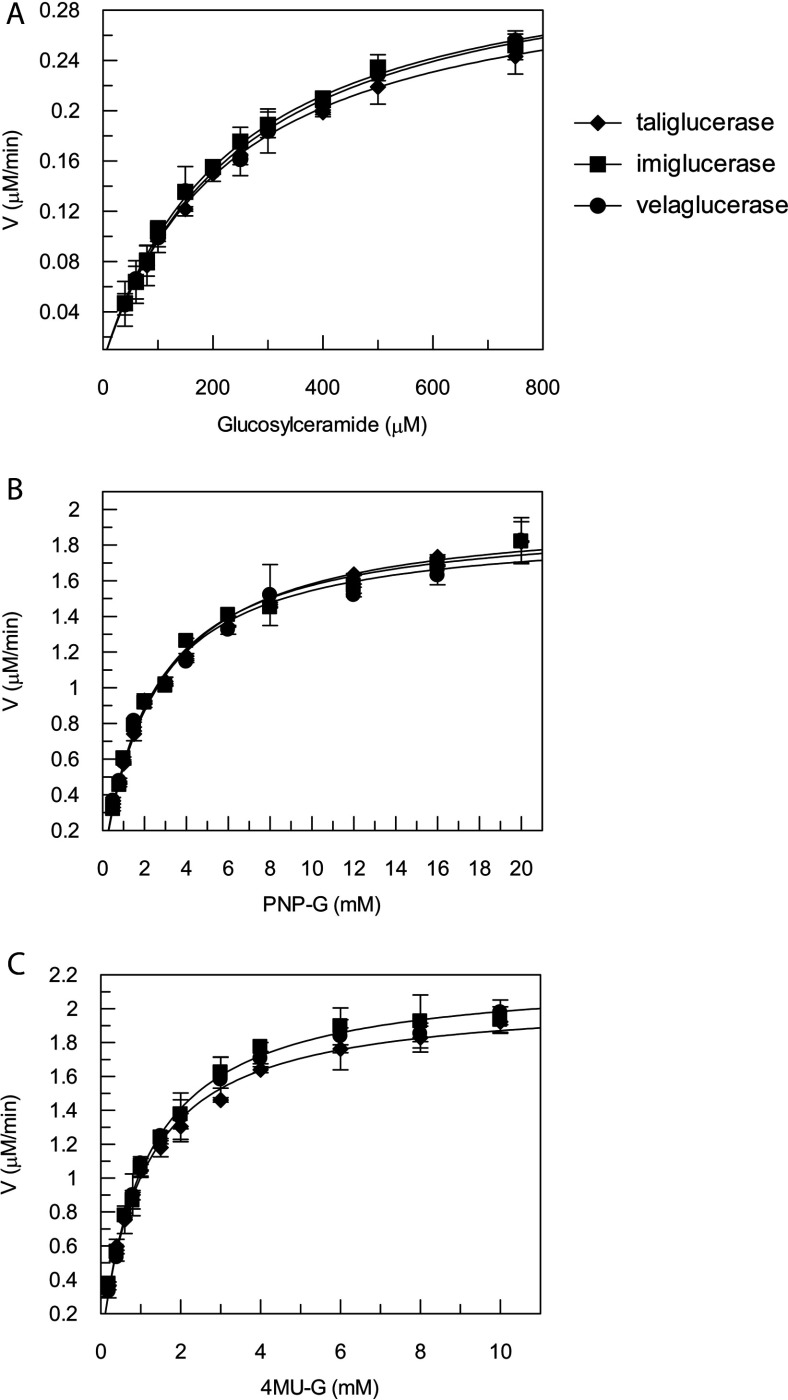
Michaelis–Menten curve Kinetic activity assay plots of velocity compared with concentration of all three β-glucocerebrosidase enzymes using (**A**) glucosylceramide and (**B**) PNP-G and (C) 4MU-G as substrates. Kinetic parameters of K_m_ and V_max_ are summarized in Table 2. Results are an average of three independent experiments ±S.D.

To understand whether the differences in glycosylation affect the biochemical properties of taliglucerase alfa, imiglucerase and velaglucerase alfa, the enzymatic activity of these three proteins was compared. Two different synthetic substrates, PNP-G and 4MU-G and the natural β-glucocerebrosidase substrate glucosylceramide were evaluated at lysosomal conditions (37°C, at pH 4.6, Figure 2A–C), and kinetic parameters were calculated (Table 2). Results show that the *in vitro* enzymatic activity of all three enzymes is similar.

**Table 2:** Kinetic parameters of β-glucocerebrosidase enzymes using three different substrates

	Taliglucerase alfa		Imiglucerase		Velaglucerase alfa	
Substrate	*K*_m_ (μM)	*V*_max_ (μM/min)	*K*_m_ (μM)	*V*_max_ (μM/min)	*K*_m_ (μM)	*V*_max_ (μM/min)
Glucosylceramide	233±12	0.321±0.007	234±11	0.336±0.007	247±14	0.338±0.008
PNP-G	2592±142	1.99±0.03	2415±165	1.96±0.04	2331±205	1.91±0.05
4MU-G	1060±52	2.07±0.05	1127±52	2.21±0.03	1107±45	2.16±0.03

**Figure 3:**
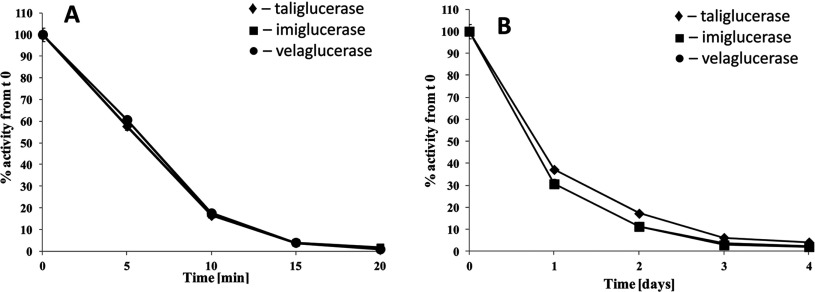
Stability in biological matrices Enzymatic stability (**A**) in plasma of healthy individuals and (**B**) at lysosomal pH (4.6) evaluated for enzymatic activity using the fluorimetric method with the synthetic substrate 4MU-G. All three plots are very similar and therefore overlap. Results are an average of three independent experiments ±S.D.

Recombinant β-glucocerebrosidase is administered intravenously. Thus, the enzyme should retain its activity in the blood circulation until taken up into its target organelle and be active in acidic pH of the lysosome. Therefore, it was important to evaluate the stability of the enzymes in either human plasma (pH 7.4) or under lysosomal conditions (pH 4.6). In both experiments, all enzymes showed a similar residual activity trend as a function of time (Figure 3). The incubation in plasma, eventually, led to protein inactivation of all enzymes after 1 h. Under lysosomal-like conditions, all enzymes showed a complete loss in activity only after 4 days.

### Cellular uptake

Glycosylation was proven to be crucial for the ability of exogenously administered β-glucocerebrosidase to be internalized into macrophages [3]. To test the glycosylation impact on cellular uptake, two cell lines were used: human macrophage-like cells, U937 [8], and a rat alveolar macrophage cell line, NR8383, as a tissue macrophage model [19].

**Figure 4:**
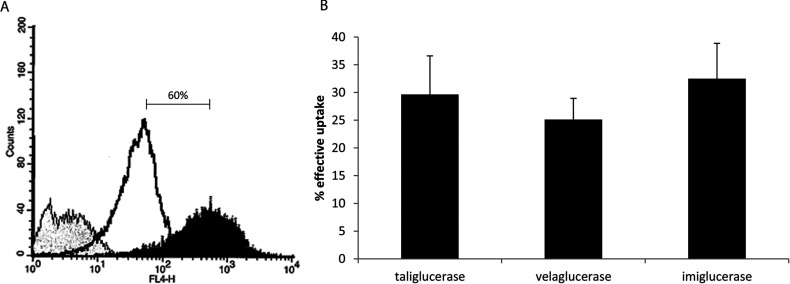
Uptake of β-glucocerebrosidase into U937 human macrophage like cell (**A**) U937 cells, treated with 75 ng/ml PMA, were stained with the macrophage marker CD11c. Unstained (negative control, grey), monocytes (untreated, open black) and macrophages (PMA treated, black) were analysed by flow cytometry. After differentiation, a 60% increase in CD11c staining can be seen; (**B**) PMA differentiated U937 macrophages were loaded with 60 μg/ml taliglucerase alfa, imiglucerase or velaglucerase alfa for 10 min at 37°C. The cells were then cooled and washed with acidic buffer. β-Glucocerebrosidase activity was tested in the cells before and after acid wash and the effective uptake is indicated. Results are the means±S.E.M. of six independent wells.

U937, a human monocytic cell line, was adopted for its ability to differentiate into macrophages [8,20]. Following differentiation with PMA, Figure 4(A) shows 60% increase of CD11c expression, a macrophage marker, on the U937 cells. After incubation with taliglucerase alfa, imiglucerase or velaglucerase alfa (60 μg/ml), enzyme activity was measured in the cell lysate. The results, represented as the effective uptake, are the ratio of the activity measured between the internalized (after acid wash) and the total (bound and internalized), β-glucocerebrosidase (Figure 4B). The uptake of all three enzymes was shown to be similar for the human macrophage-like cells (Figure 4B).

**Figure 5:**
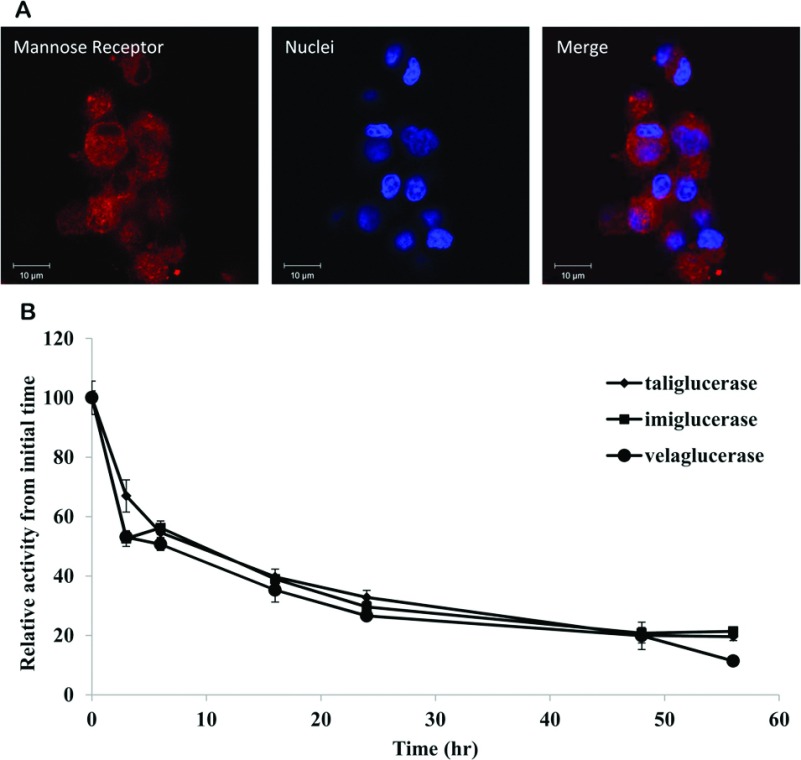
Uptake of β-glucocerebrosidase into rat alveolar macrophage cell line (**A**) Rat alveolar macrophage cell line NR8383 was fixed and stained with polyclonal anti-MR antibody produced in rabbit (1 mg/ml, Abcam), using donkey anti-rabbit conjugated to Cy3 (indocarbocyanine; Alexa Fluor) as the detection antibody. Nuclei were stained with DAPI. Pictures were taken by LSM Meta Confocal Microscopy, with a ×63 objective; (**B**) In-cell stability of taliglucerase alfa, imiglucerase or velaglucerase alfa. Enzymes were loaded into the cells at 60 μg/ml for two h. The cells were then washed and incubated up to 56 h. At each indicated time point, a sample was taken to assess the β-glucocerebrosidase activity within the cells. Results were normalized to the initial concentration of β-glucocerebrosidase in the cells. Results are means±S.E.M. of four independent wells.

To assess intracellular stability, the rat alveolar macrophage cell line (NR8383) was employed. Expression MR was established by immunofluorescence (Figure 5A). Following an initial incubation of 2 h with taliglucerase alfa, imiglucerase or velaglucerase alfa, the intracellular stability of the enzymes was determined by measuring the enzymatic activity in rat alveolar macrophages as a function of time (up to 56 h). Results show that similar intracellular stability was observed for all enzymes (Figure 5B).

### Biodistribution into organs

**Figure 6:**
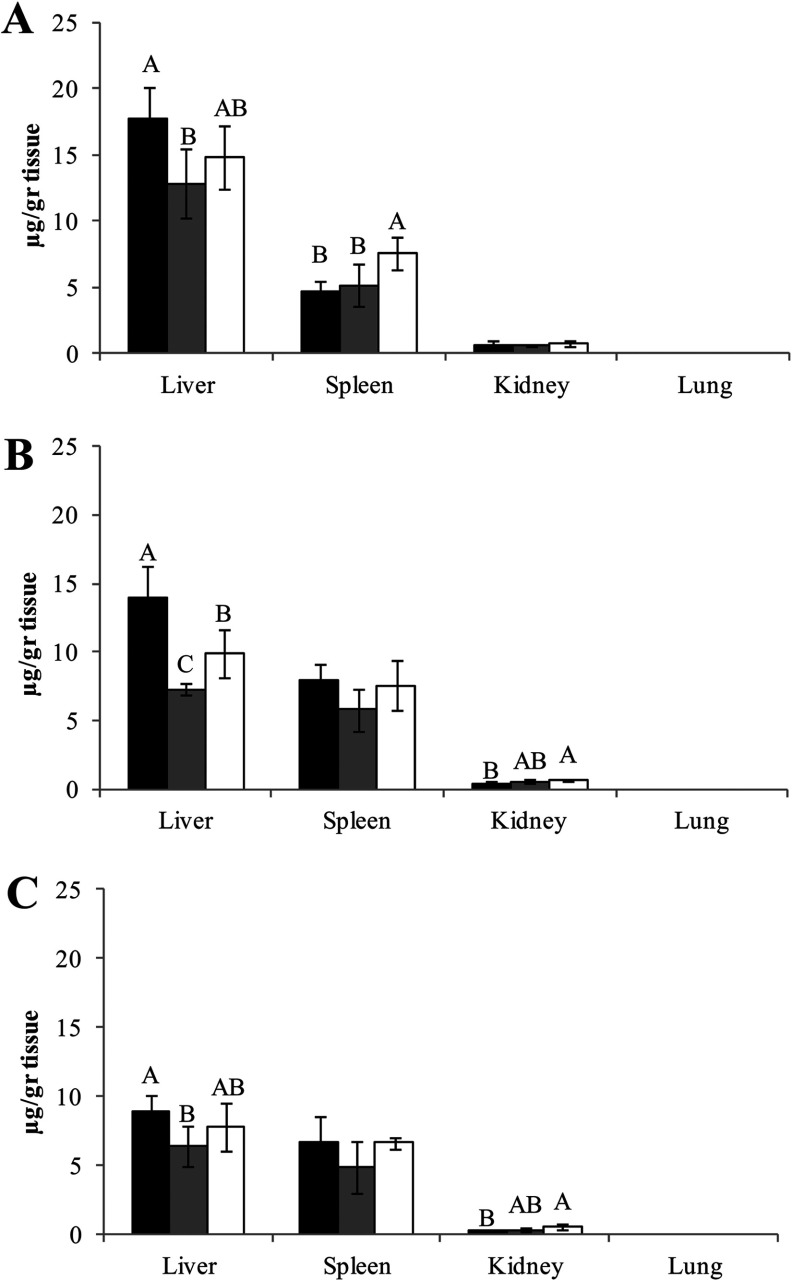
Uptake of β-glucocerebrosidase into mice organs Biodistribution to liver, spleen, kidney and lung of mice evaluated by activity measurement after: (**A**) 20; (**B**) 40; and (**C**) 60 min post-injection. ICR mice were intravenously administrated with three β-glucocerebrosidase commercial enzymes (1.8 mg/kg, similar to clinical dose in humans); taliglucerase alfa, dark bars (■); imiglucerase, grey bars (■); and velaglucerase alfa, clear bars (□). Each error bar represents the S.D. about the mean. Bars marked with the same or no letter (i.e., A, B, C) indicate that differences between results are insignificant, using analysis of one-way ANOVA Tukey–Kramer HSD. Different letters symbolize significant difference (*P*<0.05).

Biodistribution of the enzymes was assessed in ICR mice, by measuring the enzyme activity in selected target organs following a single injection of the three commercial preparations of β-glucocerebrosidase at doses of 1.8 (Figure 6) and 5 mg/kg (results not shown). Enzymatic activity was evaluated as a function of time. Similar trends were observed for all enzymes, with predominant uptake into liver and spleen, the GD target organs, as opposed to the kidney and lung (under limit of detection). A slightly higher uptake to liver was shown for taliglucerase alfa at all time points. For spleen, velaglucerase alfa uptake was higher 20 min post-injection, but this difference became non-significant as a function of time. In addition, higher uptake of velaglucerase alfa was observed in the kidney compared with taliglucerase alfa.

## DISCUSSION

This study is the first attempt to evaluate the impact of the different glycosylation profiles on the performance of the three commercially available recombinant β-glucocerebrosidase enzymes, utilizing the same analytical methods. The importance of glycosylation on the effectiveness of ERT for GD has been clearly established [4]. While all three enzymes share the same four occupied *N*-linked glycosylation sites, this study identifies a different glycan ‘fingerprint’ for taliglucerase alfa, imiglucerase and velaglucerase alfa [8,9,21]. Using a method that enables assessment of the relative amount of each glycan present, major differences were determined in the structure of the glycans (Figure 1), due to the intrinsic characteristics of the different host cells and production processes.

Based on the MS measurement of the intact proteins we were able to calculate the average glycan occupancy for the three enzymes. The molecular mass of velaglucerase alfa was found to be higher than that of taliglucerase alfa and imiglucerase, and is an outcome of the higher glycan structures found in the glycan analysis. Using this data together with the calculated mass of the amino acid composition, it was possible to calculate the theoretical glycan occupancy for each enzyme. The results show that all enzymes have the same amount of glycosylation sites occupied. These results are also in agreement with the expected, four regularly occupied glycosylation sites [11,22].

In GD, glycosphingolipid-laden macrophages, referred to as Gaucher cells [2], accumulate in the visceral tissues (liver, spleen and bone marrow). Previous studies determined that the main route of exogenous β-glucocerebrosidase internalization to macrophage cells is through the MR [3,23–25]. The specificity of this receptor to various carbohydrates has been defined with the following decreasing order of affinity, Man~Fuc>GlcNAc>Glu>>Gal [24]. Based on this knowledge, different manufacturing processes were used to produce recombinant proteins that would mainly contain exposed mannose structures.

The present study shows that the glycans in imiglucerase contain mainly a paucimannose structure substituted with a core (α1-6)-linked fucose, as expected by the process of consecutive exoglycosidase digestions [25]. In addition, this enzyme contains undigested structures with terminal GlcNAc, Gal and SA. Velaglucerase alfa glycosylation profile, obtained by addition of kifunensine to the medium [26], includes a primary glycoform of high mannose Man_9_GlcNAc_2_ that constitutes about 50% of the total glycan pool as well as multiple other glycoforms of high-mannose type. Taliglucerase alfa has a highly homogeneous glycan profile of almost 100% paucimannose structures that include an (α1-3)-linked fucose attached to the reducing GlcNAc and a xylose substituted to the bisecting mannose. Table 1 summarizes the glycan structures found in this study. As indicated, both taliglucerase alfa and velaglucerase alfa carry about 100% exposed mannose residues (paucimannose and high mannose, respectively), while imiglucerase has additional terminal structures such as GlcNAc, Gal and SA. In addition, taliglucerase alfa and imiglucerase have mainly paucimannose type glycans, while velaglucerase alfa has mostly high-mannose type glycans.

Despite the observed differences in glycosylation profile, all three recombinant β-glucocerebrosidases exhibit comparable enzymatic activity, stability and macrophage uptake capacity. This stands in agreement with the results found by Van Patten et al. [13], in which no effect on macrophage uptake, intercellular half-life and *in vivo* targeting in a GD mouse model (D409V/null) could be attributed to the various chain lengths. The only effect observed by Van Patten et al. [13] was that the longer mannose structures, similar to the ones present in velaglucerase alfa, tend to bind more efficiently to undesirable, non-MR targets, such as MBL.

Finally, uptake into target organs after intravenous administration in mice demonstrated similar results for all enzymes. The enzymatic biodistribution was evaluated at 1.8 mg/kg, corresponding to the clinical dose of 60 U/kg and 5 mg/kg. As expected, the major portion of the administered protein is delivered to the liver and spleen with slight differences in the uptake efficiency.

Macrophages are a subset of differentiated monocytes [27,28]. This differentiation causes a few changes in the structure and function of the cells, such that macrophages have phagocytic ability and express a different set of receptors on the cell surface [29]. In the present study, the uptake of the enzymes was analysed using a human monocyte cell line that was differentiated into macrophage cells (Figure 4A), the target cells for β-glucocerebrosidase. Our results show similar uptake levels of the enzymes into macrophages, supporting that differences in glycosylation have no effect on uptake to macrophage cells. Previously, Berger et al. [12] demonstrated a lower efficiency of uptake for taliglucerase alfa relative to imiglucerase and velaglucerase alfa in monocytes isolated from patients with GD. The difference in the observations may be attributed to the different nature of the cells used.

Differences in glycosylation do not seem to affect enzyme activity, substrate recognition, stability, uptake into macrophages or uptake to organs. Currently available clinical efficacy data derived from clinical trials suggest that there are no significant differences between the products in the achievement of clinical improvement in all main parameters of GD [30–32]. The availability of treatment alternatives is a valuable contribution to the management of serious diseases such as GD.

## Online data

Supplementary data
