# Supplementary material for: Glycosylation and functionality of recombinant β-glucocerebrosidase from various production systems
Source: Biosci Rep. 2013 Sep 25;33(5):e00071. doi: 10.1042/BSR20130081 (PMC3782720; doi:10.1042/BSR20130081)
Supplement: Supplementary data [file bsr033e071add.pdf]

Biosci. Rep. (2013) / **33** / art:e00071 / doi 10.1042/BSR20130081

OPEN ACCESS

Original Paper

SUPPLEMENTARY DATA

Glycosylation and functionality of recombinant β-glucocerebrosidase from various production systems

Yoram TEKOAH*[1], Salit TZABAN*, Tali KIZHNER*, Mariana HAINRICHSON*, Anna GANTMAN*, Myriam GOLEMBO*, David AVIEZER*† and Yoseph SHAALTIEL*

*Protalix Biotherapeutics, 2 Snunit Street, P. O. Box 455, Carmiel, 2161401, Israel, and †Faculty of Life Sciences, Bar Ilan University, Ramat Gan, 5290002, Israel

Supplementary Table S1 is on the following page

[1] To whom correspondence should be addressed (email yoram.tekoah@protalix.com).

© 2013 The Author(s) This is an Open Access article distributed under the terms of the Creative Commons Attribution Licence (CC-BY) (http://creativecommons.org/licenses/by/3.0/) which permits unrestricted use, distribution and reproduction in any medium, provided the original work is properly cited.

Bioscience Reports www.bioscirep.org

Table S1 Summary of relative area of peaks from NP-HPLC analysis and molecular mass detection of glycans released from taliglucerase alfa, imiglucerase and velaglucerase alfa

Fc, core fucose; G, galactose; GU, glucose units; Man_2–Man_9, $Man_{2\text{-}9}GlcNAc_2$; ND = not detected; X, xylose. Numbers are percentage areas 2% and above limit of quantitation. Molecular mass of glycans is calculated as the monisotopic mass + Na^+ adduct. Peak numbers are prefixed with T, Taliglucerase alfa, I, Imiglucerase alfa, V, Velaglucerase alfa. *, non-assigned peak.

			Taliglucerase alfa			Imiglucerase			Velaglucerase alfa		
**GU value**	**Glycan acronym**	**Mass calculated**	**Peak**	**Mass detected**	**% Area**	**Peak**	**Mass detected**	**% Area**	**Peak**	**Mass detected**	**% Area**
**4.40**	**Man3**	**933.318**	**T1**	933.577	ND	**I1**	933.940	**5**			
**4.62**	**Fc(3)Man2X**	**1049.365**	**T2**	1049.483	**2**						
**4.88**	**Fc(6)Man3 A1**	**1079.375**<br>**1136.397**				**I2**	1079.787 ND	**61**			
**4.93**	**Man3X**	**1065.360**	**T3**	1065.501	**5**						
**5.31**	**Fc(3)Man3**	**1079.375**	**T4**	ND	ND						
**5.36**	**Fc(6)A1 A2**	**1282.455**<br>**1339.476**				**I3**	1283.006 ND	**14**			
**5.84**	**Fc(6)A2**	**1485.534**				**I4**	1486.101	**5**			
**5.90**	**Fc(3)Man3X**	**1211.418**	**T5**	1211.565	**93**						
**6.16**	**Man5**	**1257.423**	**T6**	ND	ND						
**6.26**	**Fc(6)A1G**	**1444.508**				**I5**	1445.288	**4**			
**6.40**	**Fc(3)A1X**	**1414.497**	**T7**	ND	ND						
**7.09**	**A2G2**	**1663.582**				**I6**	ND	**5**			
**7.10**	**Man6**	**1419.476**	**T8**	ND	ND				**V1**	1419.117	**2**
**7.73**	**Fc(6)A2G2**	**1809.640**				**I7**	ND	**3**			
**7.85**	**Man7**	**1581.529**							**V2**	1581.114	**5**
**7.90**	*					**I8**	ND	**3**			
**8.00**	**Man7**	**1581.529**	**T9**	ND	ND				**V3**	1581.114	**6**
**8.30**	*								**V4**	ND	ND
**8.49**	*								**V5**	ND	ND
**8.72**	**FcMan7 (?)**<br>**Man8**	**1727.135**<br>**1743.581**							**V6**	1727.135<br>1743.124	**7**
**8.75**	**Man8**	**1743.581**							**V7**	1743.124	**12**
**8.90**	**Man8**	**1743.581**	**T10**	ND	ND				**V8**	1743.124	**10**
**9.12**	*								**V9**	ND	**4**
**9.55**	**Man9**	**1905.634**	**T11**	ND	ND				**V10**	1905.140	51
**10.16**	*								**V11**	ND	ND
**10.36**	*								**V12**	ND	**4**

Received 17 July 2013/6 August 2013; accepted 7 August 2013

Published as Immediate Publication 28 August 2013, doi 10.1042/BSR20130081

© 2013 The Author(s) This is an Open Access article distributed under the terms of the Creative Commons Attribution Licence (CC-BY) (http://creativecommons.org/licenses/by/3.0/) which permits unrestricted use, distribution and reproduction in any medium, provided the original work is properly cited.
